# Colorectal Cancer Risk Reduction following Macrogol Exposure: A Cohort and Nested Case Control Study in the UK

**DOI:** 10.1371/journal.pone.0083203

**Published:** 2013-12-20

**Authors:** Rachel A. Charlton, Julia M. Snowball, Katherine Bloomfield, Corinne S. de Vries

**Affiliations:** 1 Department of Pharmacy and Pharmacology, University of Bath, Bath, United Kingdom; 2 Department of Geriatric Medicine, University of Auckland, Auckland, New Zealand; The University of Chicago, United States of America

## Abstract

**Background and Aims:**

Animal studies have demonstrated macrogol laxatives may reduce colorectal cancer (CRC) risk. This study aimed to investigate the association between macrogol prescribing and CRC risk.

**Methods:**

A case-control study nested within a cohort of laxative users was conducted using data from the UK General Practice Research Database. Six controls per case were identified and to account for the lead time of CRC, additional control sets were selected on the index date backdated by 1 to 5 years. Exposure to macrogols and covariate status before each of the backdated index dates was established. Conditional logistic regression was used to calculate the risk of CRC following macrogol prescribing adjusted for potential confounders.

**Results:**

4734 incident CRC cases were identified; 2722, 2195, 1789, 1481 and 1214 had received a laxative prescription before the index dates backdated by 1 to 5 years. A suggestion of a non-significant reduction in CRC risk associated with ‘macrogol after other laxative’ prescribing was observed when the index date was backdated by 1 and 2 years, OR_adj_ = 0.87 (CI_95_0.74–1.03) and OR_adj_ = 0.80 (CI_95_0.65–1.00) compared to non-macrogol laxative exposure. The odds ratios reduced further and were significant when backdated by 3, 4 and 5 years, OR_adj_ = 0.68 (CI_95_0.50–0.92), OR_adj_ = 0.60 (CI_95_0.40–0.90) and OR_adj_ = 0.30 (CI_95_0.14–0.64) respectively. This reduction in risk was not observed, however, for ‘macrogol only’ and ‘macrogol before other laxative’ exposure categories.

**Conclusions:**

In this study we observed a reduced CRC risk associated with macrogol prescribing after accounting for the lead time for CRC. Further studies are required to determine whether the association is causal and whether it can partly be explained by selective prescribing.

## Background

Colorectal cancer is a common malignancy and a leading cause of morbidity and mortality [1]. In 2002 around one million individuals were diagnosed with the disease worldwide [2]. A large percentage of colorectal cancers develop within pre-existing adenomatous polyps or adenomas, a process which is estimated to take between five and ten years [1].

In recent years research has been carried out into the potential of chemoprevention of colorectal adenomas and colorectal cancer with the most commonly studied agents being non-steroidal anti-inflammatory drugs (NSAIDs), including aspirin [3], [4]. Initially studies focussed on high risk individuals before moving on to studies in the general population. There is now evidence from both randomised controlled trials [5]–[7] and epidemiological studies [8] suggesting that NSAIDs are chemopreventive agents, with long term exposure being associated with a reduced risk of both colorectal adenomas and colorectal cancer.

Polyethylene glycols (PEG) are inert polymers that have water absorbing properties and act as pure osmotic agents. Research over the last decade has shown PEG to be potent inhibitors of carcinogenesis in mice and rats [9]–[11]. The precise mechanism of action is still unclear although it has been proposed it may be due to the suppression of cell proliferation and the induction of apoptosis of (pre)cancerous cells, possibly as a consequence of PEG's ability to inhibit the expression and function of mucosal epidermal growth factor receptor (EGFR) [11]–[14]. In general, at the cellular level, the majority of chemo-preventative agents increase apoptosis and/or inhibit proliferation; both of these actions have been established for PEG *in vitro* and *in vivo* [12], [15]. PEG facilitates these effects via inhibition of EGFR-mediated Snail expression, leading to a concomitant increase in E-cadherin levels [11], [14]. This increased expression of E-cadherin results in an inhibition of β-catenin mediated expression of Cyclin-D1, a pathway which has been established as central to early events associated with colon carcinogenesis. A role for PEG in the induction of the cell-cycle regulator p21 has also been established [16]. Finally, it has been hypothesised that, because lipid rafts have been shown to play a role in EGFR signalling, it may be that the physiochemical properties of PEG allow for an interaction with, and the disruption of, these structures. This would then lead to impairment in the assembly and translocation of functional EGFR, ultimately resulting in inhibition of downstream signalling [17]. Study findings in mice and rats have demonstrated, however, that the suppressive effect of PEG is reversible and that the prevention of aberrant crypt foci stops when treatment is discontinued [10].

PEG, commonly referred to as macrogols, have many applications including use as an osmotic laxative which are used for treating the symptoms of chronic constipation and as bowel cleansing preparations in higher doses [18]. Given the chemoprevention potential demonstrated in rats and mice it was considered worth investigating the role of macrogols as a chemopreventive agent of colorectal cancer in humans. To our knowledge only one study has attempted to evaluate the chemopreventive potential of macrogol laxatives in humans [19]. In this population based cross-sectional study findings suggestive of a protective effect of one macrogol-4000 based laxative were observed, however no consideration was given to the timing of macrogol exposure in relation to disease initiation or to the duration or quantity of macrogol use [19]. A randomised clinical trial of the effect of macrogols on colorectal cancer risk has since been initiated, the results of which are expected towards the end of 2014 [20]. The study of colorectal cancer risk and laxative exposure is complicated by the long lead time for development of colorectal cancer. In addition a change in bowel habit is often one of the first presenting symptoms of the disease, individuals are likely to use laxatives on an ‘as needed’ basis and they may switch between products. We designed a case-control study, nested within a cohort of laxative users, which aimed to evaluate the association between macrogol laxative exposure and the risk of colorectal cancer, whilst taking these confounding factors into account.

### Ethics statement

The General Practice Research Database (GPRD) has a single Multi-Centre Ethics approval for all observational studies using GPRD data.

## Methods

### Study design

The study used data from the General Practice Research Database (GPRD) and was a cohort study of laxative users with a case-control study nested within this cohort to reduce the risk of confounding by indication. The study period ran from 1 January 2000 until 18 February 2009.

### Data source

The GPRD is a computerised database containing anonymised longitudinal data collected from UK general practice and consists largely of coded data entered by general practitioners (GPs) as part of the clinical management of patients. It is widely used for epidemiology and drug safety research [21]. When a patient visits his or her GP, the date and type of consultation is recorded along with any symptoms and clinical diagnoses, detailed prescription data and some results of clinical investigations and tests. The recording of data from each GP practice is subject to quality control checks and the database provider, the Medicines and Healthcare products Regulatory Agency (MHRA), assigns each practice an ‘up-to-standard’ (UTS) date which is the date the database provider considered the practice to have started contributing data that is of a standard suitable for the purposes of research. At the time of the study the GPRD contained over 57 million person years of data and was actively collecting data on approximately 4 million patients (∼7% of the UK population) registered at around 500 GP practices within the UK [22].

### Study population

The laxative user cohort consisted of all GPRD patients permanently registered at, or transferred out of, a GP practice providing data that the MHRA considered to be UTS for the purposes of research and who had received at least one laxative prescription during the time period their data was considered to be UTS. Laxative products were those classified under chapter 1.6.1–1.6.5 of the British National Formulary. These included all stimulant, osmotic and bulk laxatives, faecal softeners and bowel cleansing preparations. All study participants were required to have at least six months of UTS data before entering the study between 1 January 2000 and 18 February 2009.

### Identification of patients with colorectal cancer

Colorectal cancer (CRC) was defined according to the International Classification of Diseases 9^th^ edition and included ICD-9 153–154.1 (inclusive). The algorithms used to identify incident cases of CRC have been described elsewhere [23]. To summarise, individuals were identified as newly diagnosed with CRC if they had one of the following:

- a CRC diagnosis code plus additional supporting evidence such as medical codes relating to colorectal surgery, colostomy bags, cancer care review, chemo- or radiotherapy, palliative care or a terminal illness;
- a ‘neoplasm of uncertain behaviour’ code of either the colon or rectum plus supporting evidence to confirm that it was malignant such as cancer morphology codes;
- a general cancer code plus supporting evidence that it was cancer of the colon or rectum such as codes relating to colorectal surgery.

Those cases with CRC secondary to other cancer were excluded. All CRC cases were identified masked to exposure status.

The CRC index date was taken as the earliest of:

- the date of first CRC diagnosis
- the date of any CRC surgery or colostomy bag records, or
- the date of any diagnostic procedure or cancer morphology records in the 6 months before the cancer or neoplasm of uncertain behaviour record.

### Identification of controls

For each CRC case, six controls were randomly identified from the same laxative cohort population matched on year of birth, sex and date of first laxative prescription ±6 months. Consideration was given during the study design phase to the bowel cancer screening programme in the UK which was piloted from 2000–2002 and rolled out on a regional basis from 2006. It was concluded that matching by practice would only be necessary if laxative prescribing patterns differed between GP practices that were and were not participating in the screening programme, and this was thought to be unlikely. Controls were required to be disease free (i.e. have not been diagnosed with CRC) on and before the index date of the case. Controls were required to have ≥6 months of UTS data before the index date of the case.

### Classification of laxative exposure

**Figure 1:**
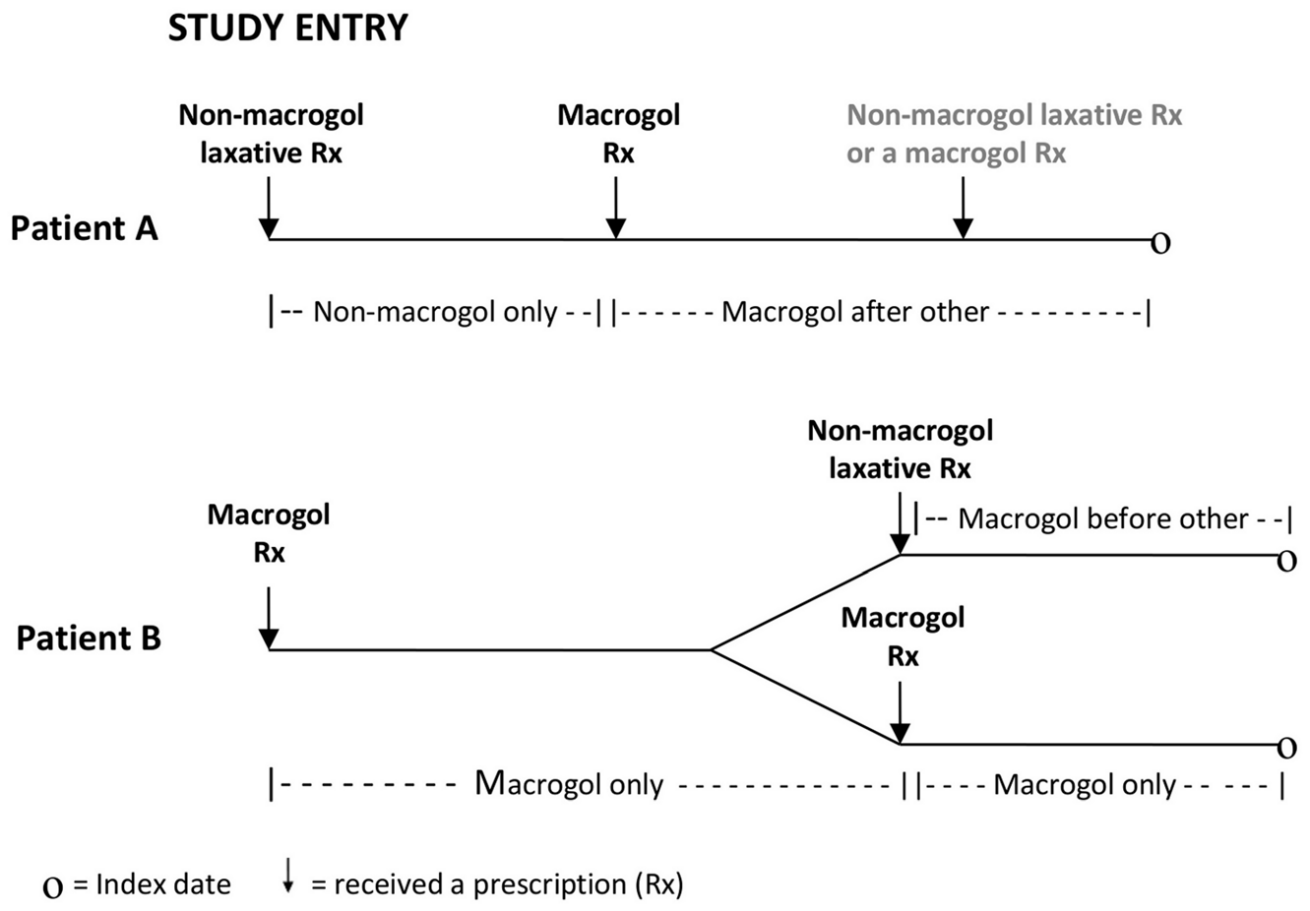
Classification of laxative exposure for those who switched laxative type.

Laxative exposure status of study participants was classified into four categories as depicted in Figure 1. Up to the point someone received their first macrogol prescription on the GPRD their person time was classified as ‘non-macrogol use only’. From the point of receiving a prescription for a macrogol their person time was classified as either ‘macrogol use only’, ‘macrogol use before other’ or ‘macrogol use after other’ as appropriate. This was done because: a) laxatives other than macrogols were not thought to cause any increase or decrease in CRC risk; b) it was unclear whether one dose of macrogol could have an impact on CRC risk, whether there was a threshold effect (a minimum number of dosages was needed to achieve a reduction in CRC risk) or whether there was a dose- or duration-response association, and c) whether, if there was a reduction in CRC risk caused by macrogol utilisation, this was because of a reduction in tumour initiation or a reduction in tumour progression. An additional ‘any macrogol’ exposure category was also created. Over the course of most of the study period, the ‘non-macrogol only’ and ‘macrogol after other’ categories would have reflected common practice in accordance with prescribing guidelines, whereas ‘macrogol only’ and ‘macrogol before other’ would have been unusual. Towards the latter years of the study, however, macrogols were more commonly being prescribed as the laxative of first choice. All laxative exposure classification was carried out masked to CRC status. For a sample of patients, free text comments recorded in addition to a medical code on the date an individual switched laxative type were requested and reviewed to provide insight into the reasons why individuals switched products.

### Power considerations

The study was powered on the case-control design. Sample size calculations were carried out using Stata and were based on an alpha of 0.05 and 80% power. Based on the assumption macrogols had 10% of the laxative market share and with the selection of 6 controls per case, to demonstrate a 20% reduction in CRC risk approximately 2,472 newly diagnosed cases were required; to demonstrate a 25% reduction in risk 1,532 cases were required. If macrogols had 20% of the market then these numbers became 1,358 and 837 respectively.

### Backdating of the colorectal cancer index date

Given the long lead time of CRC, exposure on the index date is irrelevant when assessing causality. Instead, establishing exposure at the time of tumour initiation or tumour progression is more pertinent. With that in mind we selected additional new sets of controls on the index date minus 6, 12, 18, 24, 30, 36, 42, 48, 54 and 60 months. For each analysis set the analyses of exposure and risk factors were carried out based on the backdated index date meaning that any changes in exposure status or risk factors during the period between the backdated index date and the original index date were ignored on the basis that these were irrelevant because the cancer was already present but had not been diagnosed yet. It was hypothesised that if macrogol laxatives did decrease CRC risk then any association found should become stronger or remain stable with backdating of the index date. This method has been used before to identify an increased risk of endometrial cancer associated with tibolone [24], later confirmed [25], and a similar method of backdating has been used in a diabetes study to evaluate an association between metformin and the incidence of prostate cancer [26].

### Identification of data on potential risk factors for colorectal cancer

Information was collected, where available, for both cases and controls for covariates that have been reported or suggested to be either a risk factor for, or have a protective effect against, CRC; this included smoking status, alcohol drinking status, body-mass-index, socioeconomic status, inflammatory bowel disease, history of prior cancer (excluding CRC and basal cell carcinoma), diabetes, cholecystectomy, prescriptions for any of the following: aspirin, non-steroidal anti-inflammatory drugs, 5-aminosalicylic acid, statins, opioids, hormone replacement therapy, calcium supplements, dantron containing laxatives. Smoking, alcohol, body mass index and socioeconomic status data were based on a patient's status on the index date and for the backdated analysis sets on the backdated index date. Socioeconomic status was based on an Index of Multiple Deprivation (IMD) score quintile at a patient level. CRC risk is related to diet and information on diet is not recorded within the GPRD. However, information on socioeconomic status and body mass index was included as proxy measures.

### Colorectal polyps, colonoscopies and sigmoidoscopies

As colorectal polyps are often precursors to CRC, the recording of colorectal polyps on the GPRD was explored with the aim that if there were sufficient data, investigations would be carried out to determine whether there was any supporting evidence to confirm or refute the findings of the CRC case-control study. However, it was recognised from the outset that the vast majority of polyps are undiagnosed and it was plausible that an existing association could not be identified on the GPRD as a result. To address the potential of confounding by indication, we also evaluated the recording of and the association with three or more colonoscopies or sigmoidoscopies.

### Statistical analyses

Survival analyses using Cox proportional hazards modelling with time dependent covariates were carried out for the cohort study to evaluate survival rates to colorectal cancer diagnosis, adjusting for age (as a continuous variable) and sex. Survival analyses were also carried out to evaluate all-cause mortality amongst macrogol users compared with those prescribed other laxatives. Study participants were classified as having been prescribed laxatives other than macrogols as their first laxative, macrogols as their first laxative, or macrogols following initial exposure to other laxatives. Once study participants had entered a macrogol exposure category, then this is where their person time was allocated, since other laxatives were not thought to be associated with an altered risk of CRC. However, before entering the macrogol exposure category it was possible for study participants to contribute person time to the ‘other laxatives only’ exposure category.

Conditional logistic regression analyses were carried out to evaluate any association between macrogol exposure and CRC risk in the nested case-control study. All covariates significant at the level of *p*<0.20 in the univariate analyses were considered for inclusion in the multivariate models. Covariates remained in the multivariate model if *p*≤0.05 or if they altered the risk estimate by more than 10%. Tests were carried out for interactions between variables and the stability of the models was assessed using post-estimation diagnostic statistics for conditional logistic regression models. Three sensitivity analyses were carried out within the case-control study with exclusions as follows:

- individuals <60 years to reduce exposure misclassification from over-the-counter (OTC) laxative use as over 60 s are eligible for free prescriptions in the UK;
- CRC cases with an index date before 2003 as early macrogol users may differ in terms of their CRC risk from those taking it in the later years when it had become a more first line treatment;
- chronic users of opioids as the aetiology of constipation in opioid users will differ from non-opioid users.

The total number of macrogol, lactulose and other laxative prescriptions issued were determined for each study participant to enable us to evaluate any dose response relationship. Statistical analyses were carried out using Stata 11.0 [27].

## Results

### Cohort study

**Figure 2:**
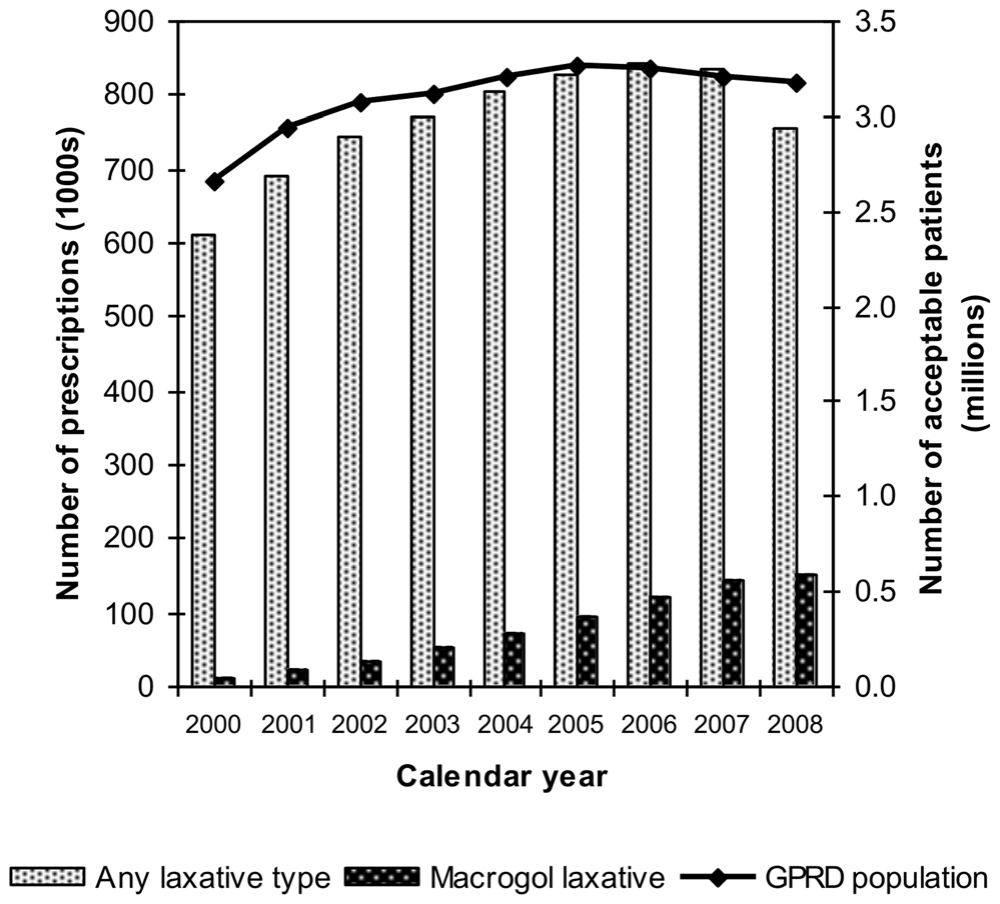
The number of all laxative prescriptions and macrogol laxative prescriptions on the GPRD by calendar year and the growth of the GPRD population.

We identified 872,959 patients on the GPRD who had received a total of almost 10 million laxative prescriptions. A total of 721,513 macrogol laxative prescriptions were identified for 155,609 individuals, of which 1,297 (0.2%) were prescriptions for bowel cleansing preparations. Figure 2 shows the number of all laxative prescriptions and macrogol laxative prescriptions on the GPRD by calendar year and the growth of the GPRD population.

Those who started on a laxative other than macrogols and then switched to PEG were less likely to develop CRC than those not receiving any macrogols at all: adjusted Hazard ratio (HR) 0.67 (CI_95_ 0.62–0.73). In contrast, those who started on a macrogol as their first laxative had a higher risk of developing CRC: adjusted HR 2.15 (CI_95_ 1.94–2.36) (Table S2). As anticipated, macrogol users who had been prescribed fewer than 21 sachets (likely associated with macrogol use just before diagnostic procedures) were more likely to receive a diagnosis than those with more macrogol exposure; HR 10.10 (CI_95_ 9.20–11.10). The strength of the association reduced with increasing exposure; HR 7.19 (CI_95_ 6.75–7.66) for 21–59 sachets and changed to a protective association, HR 0.23 (CI_95_ 0.17–0.31), in those prescribed >60 sachets. Evaluation of all-cause mortality within the cohort study found survival was better amongst those laxative users exposed to macrogols if they were preceded by other laxatives; adjusted HR 0.85 (CI_95_ 0.84–0.86) but worse amongst macrogol users whose first recorded laxative was a macrogol, compared with use of other laxatives only (adjusted HR 1.19; CI_95_ 1.17–1.21).

### Case-control study

We identified 4,734 eligible incident cases of CRC and 28,404 matched controls. Of these, 49.3% were male and 50.7% were female. The mean age at diagnosis was 73.0 years (Standard deviation (SD) = 10.9) and 74.9 years (SD = 12.2) for males and females respectively. Table 1 shows the patient characteristics of cases and controls based on information on the index date and the unadjusted odds ratios for each of the covariates in terms of CRC risk. No cases were orphaned in any of the sets.

**Table 1:** Patient characteristics for cases and controls on the index date.

		Cases		Controls		Crude odds ratio	
		N	(%)	N	(%)	(95% CI)	
**Sex**	**Males**	2,336	(49.4)	14,016	(49.4)		
	**Females**	2,398	(50.6)	14,388	(50.6)		
**Age category (years)**	**<60**	537	(11.3)	3,222	(11.3)		
	**60–64**	389	(8.2)	2,334	(8.2)		
	**65–69**	545	(11.5)	3,270	(11.5)		
	**70–74**	691	(14.6)	4,146	(14.6)		
	**75–79**	889	(18.8)	5,334	(18.8)		
	**80–84**	862	(18.2)	5,172	(18.2)		
	**85–107**	821	(17.3)	4,926	(17.3)		
**Exposure**	**Non-macrogol only**	3,755	(79.3)	23,993	(85.5)		Reference
	**Macrogol after other**	543	(11.5)	2,019	(7.1)	1.77	(1.60–1.97)
	**Macrogol only**	284	(6.0)	1,715	(6.0)	1.06	(0.92–1.22)
	**Macrogol before other**	152	(3.2)	677	(2.4)	1.43	(1.19–1.71)
**Smoking status**	**Non-smoker**	2,319	(50.0)	14,648	(51.6)		Reference
	**Smoker**	711	(15.0)	4,149	(14.6)	1.09	(0.99–1.19)
	**Ex-smoker**	1,565	(33.1)	8,802	(31.0)	1.13	(1.05–1.22)
	**Unknown**	139	(2.9)	805	(2.8)	1.09	(0.90–1.32)
**Alcohol status**	**Teetotal**	638	(13.5)	4,682	(16.5)	0.79	(0.72–0.87)
	**Drinks alcohol**	3,029	(64.0)	17,646	(62.1)		Reference
	**Heavy drinker**	110	(2.3)	589	(2.1)	1.09	(0.89–1.35)
	**Ex-drinker**	382	(8.1)	2,281	(8.0)	0.97	(0.87–1.09)
	**Unknown**	575	(12.1)	3,206	(11.3)	1.04	(0.94–1.15)
**BMI category**	**<20**	362	(7.6)	1,775	(6.2)	1.14	(1.00–1.29)
	**20–24**	1,498	(31.6)	8,319	(29.3)		Reference
	**25–29**	1,508	(31.8)	9,402	(33.1)	0.89	(0.82–0.96)
	**30–34**	545	(11.5)	3,820	(13.5)	0.79	(0.71–0.87)
	**>34**	200	(4.2)	1,402	(4.9)	0.78	(0.67–0.95)
	**Unknown**	621	(13.1)	3,686	(13.0)	0.94	(0.85–1.05)
**Socioeconomic status** *	**Quintile 1**	548	(11.6)	3,437	(12.1)		Reference
	**Quintile 2**	545	(11.5)	3,111	(11.0)	1.10	(0.97–1.25)
	**Quintile 3**	461	(9.7)	2,784	(9.8)	1.04	(0.91–1.19)
	**Quintile 4**	392	(8.3)	2,497	(8.8)	0.98	(0.86–1.13)
	**Quintile 5**	300	(6.3)	1,830	(6.4)	1.03	(0.88–1.20)
	**Unknown**	2,488	(52.6)	14,745	(51.9)	1.06	(0.96–1.17)
**Diagnosis of**	**Inflammatory bowel disease**	64	(1.4)	158	(0.6)	2.48	(1.85–3.34)
	**Type 2 diabetes**	599	(12.7)	3,539	(12.5)	1.02	(0.93–1.12)
	**Cholecystectomy**	268	(5.7)	1,802	(6.3)	0.89	(0.78–1.01)
	**Prior cancer** **	884	(18.7)	4,208	(14.8)	1.32	(1.22–1.43)
**Prescription(s) for**	**Low dose aspirin (<300 mg)**	1,805	(38.1)	12,001	(42.3)	0.82	(0.77–0.88)
	**Non-low dose aspirin (≥300 mg)**	220	(4.7)	1,560	(5.5)	0.84	(0.72–0.97)
	**5-ASA**	45	(1.0)	258	(0.9)	1.05	(0.76–1.44)
	**COX-2 inhibitors**	610	(12.9)	4,154	(14.6)	0.86	(0.78–0.94)
	**Non-selective NSAIDs <13 Rxs**	2,091	(44.2)	12,508	(44.0)	0.92	(0.86–0.98)
	**Non-selective NSAIDs ≥13 Rxs**	594	(12.6)	4,571	(16.1)	0.71	(0.64–0.78)
	**Statins**	1,290	(27.3)	8,427	(29.7)	0.87	(0.81–0.94)
	**Calcium supplements**	221	(4.7)	1,521	(5.4)	0.86	(0.74–1.00)
	**HRT** *** **≤50Rxs**	376	(7.9)	2,468	(8.7)	0.87	(0.75–0.99)
	**HRT** *** **>50 Rxs**	21	(0.4)	129	(0.5)	0.93	(0.58–1.48)
	**Opioids**	1,133	(23.9)	7,181	(25.3)	0.92	(0.86–1.00)
	**Dantron**	370	(7.8)	2,076	(7.3)	1.08	(0.96–1.22)

* Quintile 1 is the least deprived and quintile 5 is the most deprived.

** other than and not related to CRC and excluding basal cell carcinoma.

*** hormone replacement therapy.

Of the 4,734 CRC cases, 1,592 (33.6%) had received their first prescription for a laxative in the 6 months leading up to the CRC index date and they were therefore excluded from the analyses on backdated index dates. Table S1 shows the number of CRC cases eligible for inclusion in each of the backdated analysis sets. Table 2 shows patient characteristics for cases and controls combined, stratified by laxative exposure, based on status 3 years before the index date. Population characteristics based on the index date can be found in the online supplement, Table S3. Further backdating of the index date from 3 to 5 years did not result in material changes to the population characteristics although the number of eligible cases continued to reduce.

**Table 2:** Patient characteristics for cases and controls combined stratified by laxative exposure category and based on status on three years before the index date.

		Non-macrogol		Macrogol after		Macrogol only		Macrogol before		Any macrogol	
		only		other laxative				other laxative			
		N	(%)	N	(%)	N	(%)	N	(%)	N	(%)
**Sex**	**Male**	5,313	(45.2)	228	(46.0)	49	(53.3)	73	(43.7)	350	(46.4)
	**Female**	6,455	(54.8)	268	(54.0)	43	(46.7)	94	(56.3)	405	(53.6)
**Age category**	**<60**	1,362	(11.6)	32	(6.5)	9	(9.8)	18	(10.8)	59	(7.8)
**(years)**	**60–64**	840	(7.1)	25	(5.0)	5	(5.4)	12	(7.2)	42	(5.6)
	**65–69**	1,315	(11.2)	41	(8.3)	6	(6.5)	10	(6.0)	57	(7.5)
	**70–74**	2,129	(18.1)	75	(15.1)	18	(19.6)	18	(10.8)	111	(14.7)
	**75–79**	2,516	(21.4)	107	(21.6)	24	(26.1)	41	(24.6)	172	(22.8)
	**80–84**	2,225	(18.9)	120	(24.2)	15	(16.3)	41	(24.6)	176	(23.3)
	**85–107**	1,381	(11.7)	96	(19.4)	15	(16.3)	27	(16.2)	138	(18.3)
**Smoking status**	**Non-smoker**	6,277	(53.3)	232	(46.8)	49	(53.3)	90	(53.9)	371	(49.1)
	**Smoker**	1,820	(15.5)	76	(15.3)	10	(10.9)	25	(15.0)	111	(14.7)
	**Ex-smoker**	3,211	(27.3)	171	(34.5)	32	(34.8)	45	(27.0)	248	(32.8)
	**Unknown**	460	(3.9)	17	(3.4)	1	(1.1)	7	(4.2)	25	(3.3)
**Alcohol status**	**Teetotal**	2,236	(19.0)	111	(22.4)	17	(18.5)	25	(15.0)	153	(20.3)
	**Drinks alcohol**	7,247	(61.6)	276	(55.7)	60	(65.2)	103	(61.7)	439	(58.1)
	**Heavy drinker**	666	(5.7)	47	(9.5)	4	(4.4)	14	(8.4)	65	(8.6)
	**Ex-drinker**	256	(2.2)	5	(1.0)	1	(1.1)	5	(3.0)	11	(1.5)
	**Unknown**	1,363	(11.6)	57	(11.5)	10	(10.9)	20	(12.0)	87	(11.5)
**BMI category**	**<20**	677	(5.8)	52	(10.5)	6	(6.5)	18	(10.8)	76	(10.1)
	**20–24**	3,516	(29.9)	149	(30.0)	26	(28.3)	39	(23.4)	214	(28.3)
	**25–29**	3,871	(32.9)	139	(28.0)	28	(30.4)	46	(27.5)	213	(28.2)
	**30–34**	1,576	(13.4)	57	(11.5)	15	(16.3)	32	(19.2)	104	(13.8)
	**>34**	529	(4.5)	28	(5.7)	4	(4.4)	5	(3.0)	37	(4.9)
	**Unknown**	1,599	(13.6)	71	(14.3)	13	(14.1)	27	(16.2)	111	(14.7)
**Socioeconomic**	**Quintile 1**	1,335	(11.3)	55	(11.1)	9	(9.8)	12	(7.2)	76	(10.1)
**Status** *	**Quintile 2**	1,177	(10.0)	48	(9.7)	10	(10.9)	18	(10.8)	76	(10.1)
**(practice level)**	**Quintile 3**	1,090	(9.3)	48	(9.7)	11	(11.7)	22	(13.2)	81	(10.7)
	**Quintile 4**	1,006	(8.6)	46	(9.3)	10	(10.9)	14	(8.4)	70	(9.3)
	**Quintile 5**	787	(6.7)	33	(6.6)	3	(3.3)	7	(4.2)	43	(5.7)
	**Unknown**	6,373	(54.2)	266	(53.6)	49	(53.3)	94	(56.3)	409	(54.2)
**Diagnosis of**	**Innflamatory BD**	121	(1.0)	1	(1.0)	1	(1.1)	0	(0.0)	2	(0.3)
	**Type 2 diabetes**	1,279	(10.9)	55	(11.1)	11	(12.0)	17	(10.2)	83	(11.0)
	**Cholecystectomy**	768	(6.5)	42	(8.5)	6	(6.5)	12	(7.2)	60	(7.9)
	**Prior cancer** **	1,325	(11.3)	100	(20.2)	19	(20.7)	25	(15.0)	144	(19.9)
**Prescription(s)**	**Aspirin <300 mg**	4,168	(35.4)	244	(49.2)	36	(39.1)	63	(37.7)	343	(45.4)
**for**	**Aspirin ≥300 mg**	825	(7.0)	34	(6.7)	5	(5.4)	11	(6.6)	50	(6.6)
	**5-ASA**	128	(1.1)	6	(1.2)	1	(1.1)	0	(0.0)	7	(0.9)
	**COX-2 inhibitors**	1,103	(9.4)	106	(21.4)	10	(10.9)	34	(20.4)	150	(19.9)
	**Non-selective NSAIDs <13 Rxs**	5,343	(45.4)	225	(45.4)	42	(45.7)	73	(43.7)	340	(45.0)
	**Non-selective NSAIDs ≥13 Rxs**	1,817	(15.4)	92	(18.6)	10	(10.9)	24	(14.4)	126	(16.7)
	**Statins**	2,141	(18.2)	127	(25.6)	28	(30.4)	43	(24.8)	198	(26.2)
	**Calcium supplements**	686	(5.8)	38	(7.7)	2	(2.2)	9	(5.4)	49	(6.5)
	**HRT** *** **≤50Rxs**	1,142	(9.7)	50	(10.1)	9	(9.8)	14	(8.4)	73	(9.7)
	**HRT** *** **>50 Rxs**	51	(0.4)	1	(0.2)	0	(0.0)	1	(0.6)	2	(0.3)
	**Opioids**	2,471	(21.0)	186	(37.5)	25	(27.2)	42	(25.2)	253	(33.5)
	**Dantron**	1,427	(12.1)	121	(24.4)	9	(9.8)	0	(0.0)	130	(17.2)
**Macrogol**	**0**	11,768	(100.0)	0	(0.0)	0	(0.0)	0	(0.0)	0	(0.0)
**prescriptions**	**1**	0	(0.0)	240	(48.4)	46	(50.0)	98	(58.7)	384	(50.9)
	**2–3**	0	(0.0)	111	(22.4)	16	(16.7)	34	(20.4)	161	(21.3)
	**≥4**	0	(0.0)	145	(29.2)	30	(31.3)	35	(21.0)	210	(27.8)
	**range [mean]**	0	(0.0)	1–57	[4.2]	1–44	[4.8]	1–63	[3.2]	1–63	[4.0]

* 1 = least deprived, 5 = most deprived.

** not related to CRC and excluding basal cell carcinoma.

*** hormone replacement therapy.

**Figure 3:**
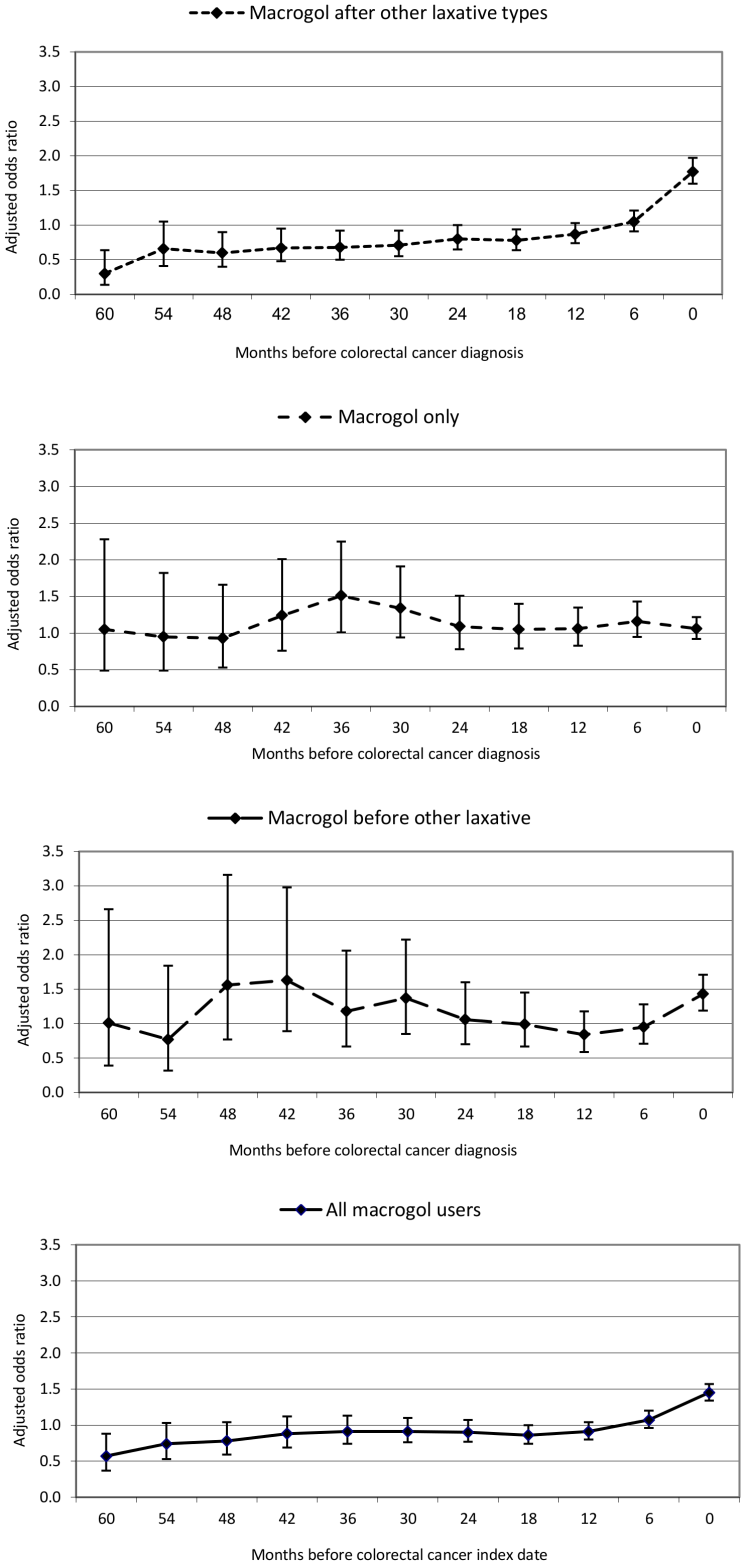
Colorectal cancer risk associated with macrogol laxative exposure compared to non-macrogol only exposure for each of the backdated analysis sets.

Table 3 and Figure 3 show the CRC risk estimates associated with the different laxative exposure categories for each of the backdated analysis sets. The risk of CRC for those individuals in the ‘macrogol after other’ category reduced with the backdating of the index date although the number of exposed CRC cases in the furthest backdated analysis sets were small. A suggestion of a non-significant reduction in CRC risk associated with ‘macrogol after other laxative’ prescribing was observed when the index date was backdated by 1 and 2 years, adjusted OR = 0.87 (CI_95_ 0.74–1.03) and adjusted OR = 0.80 (CI_95_ 0.65–1.00) compared to non-macrogol laxative exposure. The odds ratios reduced further and were significant when backdated by 3, 4 and 5 years, adjusted OR = 0.68 (CI_95_ 0.50–0.92), adjusted OR = 0.60 (CI_95_ 0.40–0.90) and adjusted OR = 0.30 (CI_95_ 0.14–0.64) respectively. This reduction in risk was not observed, however, for individuals in the ‘macrogol only’ and ‘macrogol before other laxative’ exposure categories. Sensitivity analyses restricting to those over 60 years of age, CRC cases from 2003 onwards and excluding chronic opioid users did not materially change the point estimates (data not shown). In the backdated analyses as opposed to the analyses on the index date, we found an increased CRC risk in those with BMI >30 (data not shown). Manual review of a sample of free text comments suggested that switching between different laxative products was down to personal preference and individuals' level of satisfaction with treatment; it did not highlight concerns surrounding confounding by indication.

**Table 3:** Colorectal cancer risk associated with macrogol laxative exposure compared to non-macrogol only exposure for each of the backdated analysis sets.

	Macrogol after other laxative			Macrogol laxative only			Macrogol before other laxative			Any macrogol exposure		
Analysis set	Exposed cases (n)	OR_adjusted_ * (95% CI)	*p* value	Exposed cases (n)	OR_adjusted_ (95% CI)	*p* value	Exposed cases (n)	OR_adjusted_ (95% CI)	*p* value	Exposed cases (n)	OR_adjusted_ (95% CI)	*p* value
**Index date**	543	1.77 (1.60–1.97)	<0.001	284	1.06 (0.92–1.22)	0.398	152	1.43 (1.19–1.71)	<0.001	979	1.45 (1.34–1.58)	<0.001
**Index date -6 m**	257	1.05 (0.91–1.21)	0.475	124	1.16 (0.95–1.43)	0.149	54	0.95 (0.71–1.28)	0.749	435	1.07 (0.95–1.20)	0.269
**Index date -12 m**	174	0.87 (0.74–1.03)	0.112	86	1.06 (0.83–1.35)	0.634	38	0.84 (0.59–1.18)	0.312	298	0.91 (0.80–1.04)	0.177
**Index date -18 m**	130	0.78 (0.64–0.94)	0.010	62	1.05 (0.79–1.40)	0.728	32	0.99 (0.67–1.45)	0.948	224	0.86 (0.74–1.00)	0.050
**Index date -24 m**	99	0.80 (0.65–1.00)	0.052	46	1.09 (0.78–1.51)	0.612	28	1.06 (0.70–1.60)	0.795	173	0.90 (0.76–1.07)	0.224
**Index date -30 m**	71	0.71 (0.55–0.92)	0.010	41	1.34 (0.94–1.91)	0.101	21	1.37 (0.85–2.22)	0.197	133	0.91 (0.75–1.11)	0.341
**Index date -36 m**	50	0.68 (0.50–0.92)	0.011	32	1.51 (1.01–2.25)	0.045	15	1.18 (0.67–2.06)	0.569	97	0.91 (0.73–1.14)	0.413
**Index date -42 m**	38	0.67 (0.48–0.95)	0.023	22	1.24 (0.76–2.01)	0.391	14	1.63 (0.89–2.98)	0.116	74	0.88 (0.68–1.14)	0.343
**Index date -48 m**	26	0.60 (0.40–0.90)	0.014	14	0.93 (0.53–1.66)	0.817	10	1.56 (0.77–3.16)	0.218	50	0.78 (0.58–1.06)	0.112
**Index date -54 m**	20	0.66 (0.41–1.05)	0.080	11	0.95 (0.49–1.82)	0.866	6	0.77 (0.32–1.84)	0.557	37	0.74 (0.52–1.06)	0.100
**Index date -60 m**	7	0.30 (0.14–0.64)	0.002	8	1.05 (0.49–2.28)	0.894	5	1.01 (0.39–2.66)	0.981	20	0.57 (0.36–0.91)	0.020

* OR = odds ratio. Covariates considered for adjustment included smoking status, alcohol drinking status, body mass index, socioeconomic status, inflammatory bowel disease, history of prior cancer (excluding CRC and basal cell carcinoma), diabetes, cholecystectomy, prescriptions for any of the following: aspirin, non-steroidal anti-inflammatory drugs, 5-aminosalicylic acid, statins, opioids, hormone replacement therapy, calcium supplements, dantron containing laxatives. Covariates were included in the final model where *p*≤0.05 or where the OR changed by >10%.

### Dose response investigation

Table 4 shows the results of the dose response analyses. CRC risk was reduced in the backdated case control sets in association with macrogol and lactulose use when compared with exposure to other laxatives. There was insufficient power to carry out subgroup analyses of the different macrogol exposure categories.

**Table 4:** Dose response association between macrogols, lactulose or other laxatives and colorectal cancer risk.

1 prescription	Analysis set	Other laxative	Lactulose	Macrogol
	**Index date**	Reference	1.07 (0.97–1.18)	1.56 (1.39–1.76)
	**Index - 6 m**	Reference	0.99 (0.87–1.12)	1.14 (0.96–1.36)
	**Index - 12 m**	Reference	0.94 (0.83–1.07)	0.86 (0.70–1.05)
	**Index - 18 m**	Reference	0.97 (0.84–1.10)	0.88 (0.71–1.10)
	**Index - 24 m**	Reference	0.92 (0.79–1.06)	0.91 (0.71–1.17)
	**Index - 30 m**	Reference	0.95 (0.81–1.10)	0.99 (0.75–1.30)
	**Index - 36 m**	Reference	0.88 (0.75–1.02)	0.84 (0.61–1.15)
	**Index - 42 m**	Reference	0.86 (0.74–1.02)	1.01 (0.73–0.85)
	**Index - 48 m**	Reference	0.97 (0.82–1.15)	0.90 (0.61–1.34)
	**Index - 54 m**	Reference	0.82 (0.69–0.98)	0.77 (0.49–1.20)
	**Index - 60 m**	Reference	0.84 (0.70–1.00)	0.63 (0.35–1.11)
**2–3 prescriptions**				
	**Index date**	Reference	1.12 (0.98–1.28	1.64 (1.37–1.98)
	**Index - 6 m**	Reference	1.11 (0.92–1.33)	1.03 (0.79–1.33)
	**Index - 12 m**	Reference	1.08 (0.89–1.32)	1.12 (0.84–1.49)
	**Index - 18 m**	Reference	0.98 (0.79–1.20)	0.89 (0.64–1.25)
	**Index - 24 m**	Reference	1.02 (0.83–1.27)	0.98 (0.68–1.41)
	**Index - 30 m**	Reference	0.84 (0.67–1.06)	0.77 (0.51–1.16)
	**Index - 36 m**	Reference	0.92 (0.73–1.17)	0.86 (0.52–1.41)
	**Index - 42 m**	Reference	0.96 (0.75–1.24)	0.63 (0.35–1.13)
	**Index - 48 m**	Reference	0.92 (0.71–1.18)	0.68 (0.34–1.33)
	**Index - 54 m**	Reference	0.91 (0.70–1.19)	0.56 (0.25–1.24)
	**Index - 60 m**	Reference	0.75 (0.56–0.99)	0.24 (0.06–1.00)
**4+ prescriptions**				
	**Index date**	Reference	0.92 (0.80–1.05)	1.11 (0.91–1.35)
	**Index - 6 m**	Reference	0.87 (0.75–0.99)	0.91 (0.72–1.15)
	**Index - 12 m**	Reference	0.81 (0.70–0.93)	0.67 (0.51–0.88)
	**Index - 18 m**	Reference	0.80 (0.68–0.93)	0.65 (0.48–0.87)
	**Index - 24 m**	Reference	0.78 (0.66–0.91)	0.60 (0.42–0.85)
	**Index - 30 m**	Reference	0.78 (0.66–0.92)	0.58 (0.39–0.87)
	**Index - 36 m**	Reference	0.75 (0.63–0.88)	0.67 (0.43–1.05)
	**Index - 42 m**	Reference	0.72 (0.61–0.86)	0.42 (0.22–0.78)
	**Index - 48 m**	Reference	0.72 (0.59–0.87)	0.32 (0.15–0.69)
	**Index - 54 m**	Reference	0.67 (0.55–0.82)	0.32 (0.13–0.80)
	**Index - 60 m**	Reference	0.72 (0.58–0.89)	0.32 (0.12–0.89)

### Colorectal polyps, colonoscopies and sigmoidoscopies

Information on colorectal polyps, colonoscopies and sigmoidoscopies was not recorded to a level of completeness that enabled us to confirm or refute any causal association between macrogol laxative exposure and CRC risk (data not shown).

## Discussion

This case-control study, nested within a cohort of laxative users, shows some evidence of a reduction in colorectal cancer risk associated with macrogol laxatives in humans. For individuals in the ‘macrogol after other laxative’ exposure category there was evidence of a reduction in risk. This was not observed for those in the ‘macrogol only’ or ‘macrogol before other laxative’ exposure groups, where the point estimates fluctuated around one and the 95% confidence intervals were large. The study was hypothesis driven. Backdating the index date will have eliminated some, but not all cases where undiagnosed CRC preceded laxative exposure; given the long lead time of CRC, five years of backdating may not be long enough for those cases diagnosed at an advanced stage.

Given the findings from studies carried out in mice and rats [9]–[11] and that within this study we were able to confirm the known reduction in CRC risk associated with NSAIDs, it is biologically plausible that this study may have demonstrated a true reduction in CRC risk associated with exposure to PEG-based macrogol laxatives. The three laxative exposure categories containing macrogols did, however, behave differently with only the ‘macrogol after other laxative’ category showing any evidence of a reduction in CRC risk. This could in part be explained by slight differences in population characteristics between the different laxative groups. In addition individuals in the ‘macrogol only’ category were more likely to have been prescribed only one prescription for a macrogol and they were less likely to have been prescribed more than three macrogol prescriptions than those in the ‘macrogol after other category’. If any effect is reversible in humans, as has been shown in a study in rats [10], then those who had a longer exposure may be expected to have a lower risk. Manual review of a sample of patients' electronic medical records indicated that patients in the ‘macrogol before other laxative’ category appeared to be having CRC symptoms (change in bowel habit, abdominal pain) for some time before being investigated and diagnosed, so backdating by 5 years for these individuals may not be long enough. Given the long lead time of CRC, the increased CRC risk observed for the exposure groups containing macrogols on the index date cannot be considered to be causal and is more likely to reflect individuals who are presenting with constipation as a symptom of their CRC or to reflect GPs prescribing bowel cleansing preparations in preparation for colonoscopies. We have been unable to identify how commonly, in the UK, such bowel cleansing preparations are obtained from GPs as opposed to in hospital. In addition, we had no data regarding the stage or site of the CRC, leaving us unable to evaluate any differences in risk between left-sided CRC (identifiable with a flexible sigmoidoscope) and right-sided CRC (for the diagnosis of which full colonoscopy with macrogol bowel cleansing preparation would have been required).

### Study strengths and limitations

#### Identification of colorectal cancer

Requiring 6 months of data before the index date should have helped to identify and exclude prevalent cases [28]. A number of verification studies have shown the recording of CRC on the GPRD to be reliable [23], [29]–[32]. Comparison of CRC incidence on the GPRD to that reported by the national cancer registries has found age at diagnosis to be very similar, but CRC incidence rates on the GPRD were slightly lower than those reported by the national cancer registries [23]. It is possible therefore that we may have missed a small number of CRC cases and as a result unidentified cases could have been selected in principle as a matched control. Given the relatively low prevalence of CRC and the fact that controls were selected randomly from the entire laxative user cohort, the likelihood of selecting an unidentified CRC case as a control is considered to be very low. All cases were identified masked to laxative exposure type to eliminate any potential observer bias.

#### Laxative exposure

To our knowledge only one study [33], also using data from the GPRD, has reported on the population demographics of laxative users and laxative utilisation patterns in the UK; the population demographics of laxative users recorded in the GPRD in our study were consistent with the study by Shafe *et al.* [33] and with what has been reported for individuals with constipation in North America [34]. Using data from the GPRD it was not possible to capture use of laxatives bought OTC and therefore some misclassification of exposure and delayed entry into the cohort will have occurred. Within the UK, macrogol laxatives are available without prescription but the sales are relatively low with the majority of individuals taking a macrogol having received a prescription. Macrogol bowel cleansing preparations (Klean-Prep® and Moviprep®) are often administered in a hospital setting, which may have resulted in some misclassification of macrogol exposure. However, in the vast majority of cases the amount of macrogol that an individual is likely to have been exposed to via bowel cleansing preparations before CRC diagnosis is relatively low. Overall macrogol misclassification is expected to be highest for one-off users and negligible for chronic users.

Exposure data in the GPRD is prospectively entered removing the possibility of recall bias. However, exposure was based on prescriptions issued and not necessarily on laxatives consumed, which will have resulted in some exposure misclassification. The anticipated impact of such misclassification is small: many patients had received more than one prescription and given that laxatives are prescribed to alleviate bothersome symptoms and the onset of action of macrogols is relatively slow, we consider it reasonable, especially for repeat prescribing, that chronic prescribing reflected chronic use.

#### Residual confounding

In this study we confirmed some, but not all risk factors for CRC. This may in part be a consequence of having nested the study in a cohort of laxative users – a population not representative of the general population and suffering from constipation. The latter is associated with some risk factors that overlap with those for CRC such as poor diet. We were also unable to capture all NSAID and aspirin exposure, since these products are widely available OTC.

In order for a risk factor for CRC to act as a confounder it would need to be associated both with the risk of CRC and the likelihood of an individual being prescribed a macrogol as opposed to another type of laxative. Given that many of the risk factors are unlikely to be associated with macrogol laxative exposure it was not surprising that adjustment for potential CRC risk factors in the logistic regression models made little difference to the point estimates. Within the GPRD there is no information on diet or physical activity, which are known CRC risk factors; however, information on BMI and socio-economic status was available and included as proxy measures. We were unable to replicate the increase in CRC risk associated with obesity and our risk estimate for the association with smoking was lower than that observed elsewhere [35]–[37]. We were unable to replicate the association with alcohol [38]. Given the fact that statistical adjustment for BMI and socio-economic status did not alter the point estimates, and given the fact that these covariates are strongly associated with diet and physical activity but not the choice of laxative, it is considered unlikely but not impossible that they acted as residual confounders. Similarly, residual confounding by smoking and alcohol status is considered unlikely but not impossible.

Review of free text data in relation to switching of laxative type did not highlight concerns over possible confounding by indication. It is understandable that individuals may choose to switch products given differences in the speed of action, preferences for either tablets or sachets and concerns over possible bowel dependence with some forms of laxative.

#### Diagnostic procedures

The recording of colonoscopies and sigmoidoscopies was incomplete in the GPRD so it was not possible to evaluate any association with macrogol exposure. Although speculative, it is possible that individuals prescribed a macrogol following a lack of success with other laxatives (OTC or prescription) were more likely to be referred for investigative procedures resulting in the detection of precancerous lesions that were subsequently removed before progressing to CRC. This would be more plausible in the early years before macrogols became a first line therapy but if it were the case an indirect higher level of investigation rather than the macrogol exposure itself could be partially responsible for the observed reduction in risk.

#### Dose response

Evaluation of dose between different types of laxative products was complicated by the fact that individuals switch between different laxatives and by the fact that in the ‘other’ categories there was more opportunity for longer duration of use because when people switched between products within the latter category, they remained within the ‘other’ category whereas when people switched from macrogols, they switched to a different exposure category altogether. However, in the cohort study, exposure to larger numbers of sachets was associated with a reduced risk. In the case-control study a reduction in CRC risk was identified both amongst lactulose and amongst macrogol users with increasing numbers of lactulose prescriptions received, when compared with other laxatives.

## Conclusions

In conclusion, this study suggests macrogol use is associated with a reduction in risk of colorectal cancer. Whether this association is causal is unclear at this stage; the fact that no association was found in those who started their laxative use with macrogols as opposed to those for whom macrogols were second line treatment suggests the results may be partly explained by selective prescribing. However, extensive data exploration and sensitivity analyses could not identify any indication of such channelling bias occurring. In our view, a randomised controlled clinical trial would provide more definitive evidence.

## Supporting Information

Table S1 **The number of cases and matched controls in each of the backdated analysis sets.**
(DOCX)

Table S2 **Survival rates to colorectal cancer and all-cause mortality for macrogol users compared to those prescribed other laxatives.**
(DOCX)

Table S3 **Patient characteristics for cases and controls combined stratified by laxative exposure category and based on status on the index date.**
(DOCX)

## References

[1] Midgley R, Kerr D (1999) Colorectal cancer. Lancet 353: 391–399. 9950460 10.1016/S0140-6736(98)07127-X

[2] Parkin DM, Bray F, Ferlay J, Pisani P (2005) Global cancer statistics, 2002. CA Cancer J Clin 55: 74–108. 15761078 10.3322/canjclin.55.2.74

[3] Langman M, Boyle P (1998) Chemoprevention of colorectal cancer. Gut 43: 578–585. 9824590 10.1136/gut.43.4.578 PMC1727277

[4] Janne PA, Mayer RJ (2000) Chemoprevention of colorectal cancer. N Engl J Med 342: 1960–1968. 10874065 10.1056/NEJM200006293422606

[5] Baron JA, Cole BF, Sandler RS, Haile RW, Ahnen D, et al (2003) A randomized trial of aspirin to prevent colorectal adenomas. N Engl J Med 348: 891–899. 12621133 10.1056/NEJMoa021735

[6] Bertagnolli MM, Eagle CJ, Zauber AG, Redston M (2009) Five-Year Efficacy and Safety Analysis of the Adenoma Prevention with Celecoxib Trial. Cancer Prev Res 2: 310–321. 10.1158/1940-6207.CAPR-08-0206 PMC2976587 19336730

[7] Rothwell PM, Wilson M, Elwin C-E, Norrving B, Algra A, et al (2010) Long-term effect of aspirin on colorectal cancer incidence and mortality: 20-year follow-up of five randomised trials. Lancet 376: 1741–1750. 20970847 10.1016/S0140-6736(10)61543-7

[8] Din FVN, Theodoratou E, Farrington SM, Tenesa A, Barnetson RA, et al (2010) Effect of aspirin and NSAIDs on risk and survival from colorectal cancer. Gut 59: 1670–1679. 20844293 10.1136/gut.2009.203000

[9] Parnaud G, Tach‚ S, Peiffer G, Corpet D (1999) Polyethylene-glycol suppresses colon cancer and causes dose-dependent regression of azoxymethane-induce aberrant crypt foci in rats. Cancer Res 59: 5143–5147. 10537289

[10] Corpet D, Parnaud G, Delverdier M, Peiffer G, Tach‚ S (2000) Consistent and fast inhibition of colon carcinogenesis by polyethylene glycol in mice and rats given various carcinogens. Cancer Res 60: 3160–3164. 10866305

[11] Roy HK, Kunte DP, Koetsier JL, Hart J, Kim YL, et al (2006) Chemoprevention of colon carcinogenesis by polyethylene glycol: supression of epithelial proliferation via modulation of SNAIL/ƒ-catenin signaling. Mol Cancer Ther 5: 2060–2069. 16928827 10.1158/1535-7163.MCT-06-0054

[12] Roy HK, DiBaise JK, Black J, Karolski WJ, Ratashak A, et al (2001) Polyethylene glycol induces apoptosis in HT-29 cells: potential mechanism for chemoprevention of colon cancer. FEBS Letters 496: 143–146. 11356199 10.1016/s0014-5793(01)02420-6

[13] Tache S, Parnaud G, Van Beek E, Corpet D (2006) Polyethylene glycol, unique among laxatives, supresses aberrant crypt foci, by elimination of cells. Scand J Gastroenterol 41: 730–736. 16716974 10.1080/00365520500380668 PMC2643349

[14] Wali RK, Kunte DP, Koetsier JL, Bissonnette M, Roy HK (2008) Polyethylene glycol-mediated colorectal cancer chemoprevention: roles of epidermal growth factor receptor and Snail. Mol Cancer Ther 7: 3103–3111. 18790788 10.1158/1535-7163.MCT-08-0434 PMC2547487

[15] Parnaud G, Corpet D, Gamet-Payrastre L (2001) Cytostatic effect of polyethylene glycol on human colonic adenocarcinoma cells. Int J Cancer 92: 63–69. 11279607 10.1002/1097-0215(200102)9999:9999<::AID-IJC1158>3.0.CO;2-8

[16] Roy HK, Koetsier JL, Tiwari AK, Joshi S, Kunte DP, et al (2011) Involvement of p21cip1/waf1 in the anti-proliferative effects of polyethylene glycol in colon carcinogenesis. Int J Oncol 38: 529–536. 21170505 10.3892/ijo.2010.875

[17] Coskin U, Grzybek M, Drechsel D, Simons K (2011) Regulation of human EGF receptors by lipids. Proc Natl Acad Sci U S A 108: 9044–9048. 21571640 10.1073/pnas.1105666108 PMC3107302

[18] Attar A, Lémann M, Ferguson A, Halphen M, Boutron M-C, et al (1999) Comparison of a low dose polyethylene glycol electrolyte solution with lactulose for treatment of chronic constipation. Gut 44: 226–230. 9895382 10.1136/gut.44.2.226 PMC1727381

[19] Dorval E, Jankowski JM, Association G, Barbieux JP, Viguier J, et al (2006) Polyethylene glycol and prevalence of colorectal adenomas. Gastroenterol Clin Biol 30: 1196–1199. 17075478 10.1016/s0399-8320(06)73511-4

[20] ClinicalTrials.gov Study identifier NCT00828984.

[21] Wood L, Martinez C (2004) The General Practice Research Database: Role in pharmacovigilance. Drug Saf 27: 871–881. 15366975 10.2165/00002018-200427120-00004

[22] MHRA (2009) The General Practice Research Database.

[23] Charlton R, Snowball J, Bloomfield K, de Vries C (2012) Colorectal cancer incidence on the General Practice Research Database. Pharmacoepidemiol Drug Saf 21: 775–783. 22383247 10.1002/pds.3236

[24] de Vries CS, Bromley SE, Thomas H, Farmer RDT (2005) Tibolone and Endometrial Cancer: A Cohort and Nested Case-Control Study in the UK. Drug Saf 28: 241–249. 15733028 10.2165/00002018-200528030-00005

[25] Million Women Study Collaborators (2005) Endometrial cancer and hormone-replacement therapy in the Million Women Study. Lancet 365: 1543–1551. 15866308 10.1016/S0140-6736(05)66455-0

[26] Azoulay L, Dell'Aniello S, Gagnon B, Pollak M, Suissa S (2010) Metformin and the Incidence of Prostate Cancer in Patients with Type 2 Diabetes. Cancer Epidemiol Biomarkers Prev 10.1158/1055-9965.EPI-10-0940 21148757

[27] StataCorp. 2009. Stata Statistical Software: Release 11. College Station TSL

[28] Lewis JD, Bilker W, Weinstein RB, Strom B (2005) The relationship between time since registration and measured incidence rates in the General Practice Research Database. Phamacepidemiol Drug Saf 14: 443–451. 10.1002/pds.1115 15898131

[29] Boggon R, van Staa TP, Chapman M, Gallagher AM, Hammad TA, et al (2013) Cancer recording and mortality in the General Practice Research Database and linked cancer registries. Pharmacoepidemiology and Drug Safety 22: 168–175. 23239282 10.1002/pds.3374

[30] Dregan A, Moller H, Murray-Thomas T, Gulliford MC (2012) Validity of cancer diagnosis in a primary care database compared with linked cancer registrations in England. Population-based cohort study. Cancer Epidemiology 36: 425–429. 22727737 10.1016/j.canep.2012.05.013

[31] Garcia-Rodriguez LA, Huerta-Alvarez C (2001) Reduced risk of colorectal cancer among long-term users of aspirin and non-aspirin non-steroidal and anti-inflammatory drugs. Epidemiology 12: 88–93. 11138826 10.1097/00001648-200101000-00015

[32] Yang YX, Hennessy S, Lewis JD (2004) Insulin therapy and colorectal cancer risk among type 2 diabetes mellitus patients. Gastroenterology 127: 1044–1050. 15480982 10.1053/j.gastro.2004.07.011

[33] Shafe ACE, Lee S, Dalrymple JSO, Whorwell PJ (2011) The LUCK study: Laxative Usage in patients with GP-diagnosed Constipation in the UK, within the general population and in pregnancy. An epidemiological study using the General Practice Research Database (GPRD). Therap Adv Gastroenterol 4: 343–363. 10.1177/1756283X11417483 PMC3187684 22043228

[34] Higgins PDR, Johanson JF (2004) Epidemiology of Constipation in North America: A Systematic Review. Am J Gastroenterol 99: 750–759. 15089911 10.1111/j.1572-0241.2004.04114.x

[35] Botteri E, Iodice S, Bagnardi V, Raimondi S, Lowenfels AB, et al (2008) Smoking and colorectal cancer: A meta-analysis. JAMA 300: 2765–2778. 19088354 10.1001/jama.2008.839

[36] Larsson SC, Wolk A (2007) Obesity and colon and rectal cancer risk: a meta-analysis of prospective studies. Am J Clin Nutr 86: 556–565. 17823417 10.1093/ajcn/86.3.556

[37] Liang PS, Chen T-Y, Giovannucci E (2009) Cigarette smoking and colorectal cancer incidence and mortality: Systematic review and meta-analysis. Int J Cancer 124: 2406–2415. 19142968 10.1002/ijc.24191

[38] Moskal A, Norat T, Ferrari P, Riboli E (2007) Alcohol intake and colorectal cancer risk: A dose–response meta-analysis of published cohort studies. Int J Cancer 120: 664–671. 17096321 10.1002/ijc.22299

