# Supplementary material for: Colorectal Cancer Risk Reduction following Macrogol Exposure: A Cohort and Nested Case Control Study in the UK
Source: PLoS One. 2013 Dec 20;8(12):e83203. doi: 10.1371/journal.pone.0083203 (PMC3869778; doi:10.1371/journal.pone.0083203)
Supplement: Table S1 — The number of cases and matched controls in each of the backdated analysis sets. (DOCX) [file pone.0083203.s001.docx]

**Table S1** The number of cases and matched controls in each of the backdated analysis sets

| **Analysis set** | **Cases (n)** | **Controls (n)** |
| --- | --- | --- |
| **Index date** | 4,734 | 28,404 |
| **Index date -6m** | 3,142 | 18,852 |
| **Index date -12m** | 2,722 | 16,332 |
| **Index date -18m** | 2,445 | 14,670 |
| **Index date -24m** | 2,195 | 13,170 |
| **Index date -30m** | 1,982 | 11,892 |
| **Index date -36m** | 1,789 | 10,734 |
| **Index date -42m** | 1,636 | 9,816 |
| **Index date -48m** | 1,481 | 8,886 |
| **Index date -54m** | 1,351 | 8,106 |
| **Index date -60m** | 1,214 | 7,284 |
