# Supplementary material for: Colorectal Cancer Risk Reduction following Macrogol Exposure: A Cohort and Nested Case Control Study in the UK
Source: PLoS One. 2013 Dec 20;8(12):e83203. doi: 10.1371/journal.pone.0083203 (PMC3869778; doi:10.1371/journal.pone.0083203)
Supplement: Table S2 — Survival rates to colorectal cancer and all-cause mortality for macrogol users compared to those prescribed other laxatives. (DOCX) [file pone.0083203.s002.docx]

**Table S2** Survival rates to colorectal cancer and all-cause mortality for macrogol users compared to those prescribed other laxatives

|  | **HR_adj_^*^** | **(95% CI)** |
| --- | --- | --- |
| **Colorectal cancer diagnosis** |  |  |
| Non-macrogol only | Reference |  |
| Macrogol after other | 0.67 | (0.62-0.73) |
| Macrogol before other | 2.15 | (1.94-2.36) |
|  |  |  |
| **All-cause mortality** |  |  |
| Non-macrogol only | Reference |  |
| Macrogol after other | 0.85 | (0.84-0.86) |
| Macrogol before other | 1.19 | (1.17-1.21) |

* adjusted for age as a continuous variable and sex
