# Supplementary material for: Colorectal Cancer Risk Reduction following Macrogol Exposure: A Cohort and Nested Case Control Study in the UK
Source: PLoS One. 2013 Dec 20;8(12):e83203. doi: 10.1371/journal.pone.0083203 (PMC3869778; doi:10.1371/journal.pone.0083203)
Supplement: Table S3 — Patient characteristics for cases and controls combined stratified by laxative exposure category and based on status on the index date. (DOCX) [file pone.0083203.s003.docx]

**Table S3** Patient characteristics for cases and controls combined stratified by laxative exposure category and based on status on the index date.

|  |  | **Non-macrogol only** | | **Macrogol after other laxative** | | **Macrogol only** | | **Macrogol before other laxative** | | **Any macrogol** | |
| --- | --- | --- | --- | --- | --- | --- | --- | --- | --- | --- | --- |
|  |  | **N** | **(%)** | **N** | **(%)** | **N** | **(%)** | **N** | **(%)** | **N** | **(%)** |
| **Sex** | **Male** | 13,575 | (48.9) | 1,241 | (48.4) | 441 | (53.2) | 1,095 | (54.8) | 2,777 | (51.5) |
|  | **Female** | 14,173 | (51.1) | 1,321 | (51.6) | 388 | (46.8) | 904 | (45.2) | 2,613 | (48.5) |
| **Age category** | **<60** | 3,205 | (11.6) | 168 | (6.6) | 92 | (11.1) | 294 | (14.7) | 554 | (10.3) |
| **(years)** | **60-64** | 2,320 | (8.4) | 124 | (4.8) | 55 | (6.6) | 224 | (11.2) | 403 | (7.5) |
|  | **65-69** | 3,285 | (11.8) | 193 | (7.5) | 98 | (11.8) | 239 | (12.0) | 530 | (9.8) |
|  | **70-74** | 4,103 | (14.8) | 319 | (12.5) | 114 | (13.8) | 301 | (15.1) | 734 | (13.6) |
|  | **75-79** | 5,173 | (18.6) | 515 | (20.1) | 167 | (20.1) | 368 | (18.4) | 1,050 | (19.5) |
|  | **80-84** | 5,001 | (18.0) | 581 | (22.7) | 149 | (18.0) | 303 | (15.2) | 1,033 | (19.2) |
|  | **85-107** | 4,661 | (16.8) | 662 | (25.8) | 154 | (18.6) | 270 | (13.5) | 1,086 | (20.1) |
| **Smoking status** | **Non-smoker** | 14,282 | (51.5) | 1,289 | (50.3) | 398 | (48.0) | 998 | (49.9) | 2,685 | (49.8) |
|  | **Smoker** | 4,110 | (14.8) | 321 | (12.5) | 134 | (16.2) | 295 | (14.8) | 750 | (13.9) |
|  | **Ex-smoker** | 8,511 | (30.7) | 899 | (35.1) | 285 | (34.4) | 672 | (33.6) | 1,856 | (34.4) |
|  | **Unknown** | 845 | (3.1) | 53 | (2.1) | 12 | (1.4) | 34 | (1.7) | 99 | (1.8) |
| **Alcohol status** | **Teetotal** | 4,505 | (16.2) | 404 | (15.8) | 139 | (16.8) | 272 | (13.6) | 815 | (15.1) |
|  | **Drinks alcohol** | 17,368 | (62.6) | 1,529 | (59.7) | 477 | (57.5) | 1,301 | (65.1) | 3,307 | (61.4) |
|  | **Heavy drinker** | 591 | (2.1) | 37 | (1.4) | 24 | (2.9) | 47 | (2.4) | 108 | (2.0) |
|  | **Ex-drinker** | 2,116 | (7.6) | 290 | (11.3) | 90 | (10.9) | 167 | (8.4) | 547 | (10.1) |
|  | **Unknown** | 3,168 | (11.4) | 302 | (11.8) | 99 | (11.9) | 212 | (10.6) | 613 | (11.4) |
| **BMI category** | **<20** | 1,735 | (6.3) | 214 | (8.3) | 69 | (8.3) | 119 | (6.0) | 402 | (7.5) |
|  | **20-24** | 8,150 | (29.4) | 824 | (32.2) | 239 | (28.8) | 604 | (30.2) | 1,667 | (30.9) |
|  | **25-29** | 9,235 | (33.3) | 765 | (29.9) | 275 | (33.2) | 635 | (31.8) | 1,675 | (31.1) |
|  | **30-34** | 3,686 | (13.3) | 310 | (12.1) | 97 | (11.7) | 272 | (13.6) | 679 | (12.6) |
|  | **>34** | 1,329 | (4.8) | 105 | (4.1) | 41 | (5.0) | 127 | (6.4) | 273 | (5.1) |
|  | **Unknown** | 3,613 | (13.0) | 344 | (13.4) | 108 | (13.0) | 242 | (12.1) | 694 | (12.9) |
| **Socioeconomic** | **Quintile 1*** | 3,308 | (11.9) | 316 | (12.3) | 106 | (12.8) | 255 | (12.8) | 677 | (12.6) |
| **status** | **Quintile 2** | 3,028 | (10.9) | 279 | (10.9) | 94 | (11.3) | 255 | (12.8) | 628 | (11.7) |
|  | **Quintile 3** | 2,709 | (9.8) | 253 | (9.9) | 86 | (10.4) | 197 | (9.9) | 535 | (9.9) |
|  | **Quintile 4** | 2,427 | (8.7) | 244 | (9.5) | 60 | (7.2) | 158 | (7.9) | 462 | (8.6) |
|  | **Quintile 5*** | 1,795 | (6.5) | 187 | (7.3) | 51 | (6.2) | 97 | (4.9) | 335 | (6.2) |
|  | **Unknown** | 14,481 | (52.2) | 1,283 | (50.1) | 432 | (52.1) | 1,037 | (51.9) | 2,752 | (51.1) |
| **Diagnosis of** | **Inflammatory bowel disease** | 194 | (0.7) | 19 | (0.7) | 4 | (0.5) | 5 | (0.3) | 28 | (0.5) |
|  | **Type 2 diabetes** | 3,341 | (12.0) | 378 | (14.8) | 130 | (15.7) | 289 | (14.5) | 797 | (14.8) |
|  | **Cholecystectomy** | 1,728 | (6.2) | 183 | (7.1) | 49 | (5.9) | 110 | (5.5) | 342 | (6.3) |
|  | **Prior cancer **** | 4,023 | (14.5) | 522 | (20.4) | 179 | (21.6) | 368 | (18.4) | 1,069 | (19.8) |
| **Prescription(s)** | **Aspirin <300mg** | 11,298 | (40.7) | 1,325 | (51.7) | 380 | (45.8) | 803 | (40.2) | 2,508 | (46.5) |
| **for** | **Aspirin ≥300mg** | 1,505 | (5.4) | 165 | (6.4) | 30 | (3.6) | 80 | (4.0) | 275 | (5.1) |
|  | **5-ASA** | 260 | (0.9) | 26 | (1.0) | 5 | (0.6) | 12 | (0.6) | 43 | (0.8) |
|  | **COX-2 inhibitors** | 3,704 | (13.3) | 596 | (23.3) | 136 | (16.4) | 328 | (16.4) | 1,060 | (19.7) |
|  | **Non-selective NSAIDs <13 Rxs** | 12,237 | (44.1) | 1,154 | (45.0) | 351 | (42.3) | 857 | (42.9) | 2,362 | (43.8) |
|  | **Non-selective NSAIDs ≥13 Rxs** | 4,322 | (15.6) | 457 | (17.8) | 114 | (13.8) | 272 | (13.6) | 843 | (15.6) |
|  | **Statins** | 7,707 | (27.8) | 976 | (38.1) | 286 | (34.5) | 749 | (37.5) | 2,011 | (37.3) |
|  | **Calcium supplements** | 1,452 | (5.2) | 192 | (7.5) | 32 | (3.9) | 66 | (3.3) | 290 | (5.4) |
|  | **HRT*** ≤50Rxs** | 2,421 | (8.7) | 203 | (7.9) | 53 | (6.4) | 167 | (8.4) | 423 | (7.8) |
|  | **HRT*** >50 Rxs** | 122 | (0.4) | 17 | (0.7) | 3 | (0.4) | 8 | (0.4) | 28 | (0.5) |
|  | **Opioids** | 6,482 | (23.4) | 948 | (37.0) | 287 | (34.6) | 597 | (29.9) | 1,832 | (34.0) |
|  | **Dantron** | 1,985 | (7.2) | 410 | (16.0) | 51 | (6.2) | 0 | (0.0) | 461 | (8.6) |
| **Macrogol** | **0** | 27,748 | (100.0) | 0 | (0.0) | 0 | (0.0) | 0 | (0.0) | 0 | (0.0) |
| **prescriptions** | **1** | 0 | (0.0) | 1,194 | (46.6) | 432 | (55.5) | 1,394 | (69.7) | 3,202 | (56.0) |
|  | **2-3** | 0 | (0.0) | 649 | (25.3) | 184 | (23.6) | 375 | (18.8) | 1,208 | (22.4) |
|  | **≥ 4** | 0 | (0.0) | 719 | (28.1) | 163 | (20.9) | 230 | (11.5) | 1,112 | (20.6) |
|  | **range (mean)** | 0 | (0.0) | 1-76 | [4.4] | 1-80 | [3.9] | 1-67 | [2.3] | 1-80 | [2.8] |

*1 = least deprived, 5 = most deprived ****** not related to CRC and excluding basal cell carcinoma *** hormone replacement therapy
